# Stress Relieving Heat Treatment of 316L Stainless Steel Made by Additive Manufacturing Process

**DOI:** 10.3390/ma16196461

**Published:** 2023-09-28

**Authors:** Matúš Gel’atko, Michal Hatala, František Botko, Radoslav Vandžura, Jiří Hajnyš, Michal Šajgalík, Jozef Török

**Affiliations:** 1 Faculty of Manufacturing Technologies, Technical University of Košice, 080 01 Prešov, Slovakia; matus.gelatko@tuke.sk (M.G.);; 2 Center of 3D Printing Protolab, Department of Machining, Assembly and Engineering Technology, Faculty of Mechanical Engineering, VSB-TU Ostrava, 17. Listopadu 2172/15, 708 00 Ostrava, Czech Republic; 3 Department of Machining and Manufacturing Technology, Faculty of Mechanical Engineering, University of Žilina, Univerzitná 1, 010 26 Žilina, Slovakia

**Keywords:** stainless steel, heat treatment, residual stress, microstructure, selective laser melting, X-ray diffraction

## Abstract

Residual stress occurs in the materials after different methods of processing due to the application of pressure and/or thermal gradient. The occurrence of residual stresses can be observed in both subtractive and additive-manufactured (AM) materials and objects. However, pressure residual stresses are considered, in some cases, to have a positive effect; there are applications where the neutral stress state is required. As there is a lack of standards describing the heat treatment of AM materials, there is a need for experimental research in this field. The objective of this article is to determine the heat treatment thermal regime to achieve close to zero stress state in the subsurface layer of additively manufactured AM316L stainless steel. The presented objective leads to the long-term goal of neutral etalons for eddy current residual stress testing preparation. A semi-product intended for the experiment was prepared using the Selective Laser Melting (SLM) process and subsequently cut, using Abrasive Water Jet (AWJ) technology, into experimental specimens, which were consequently heat-treated in combination with four temperatures and three holding times. Residual stresses were measured using X-ray diffraction (XRD), and microstructure variations were observed and examined. A combination of higher temperature and longer duration of heat treatment caused more significant stress relaxation, and the original stress state of the material influenced a degree of this relaxation. The microstructure formed of cellular grains changed slightly in the form of grain growth with randomly occurring unmolten powder particles, porosity, and inclusion precipitation.

## 1. Introduction

Nowadays, additive manufacturing (AM) is changing the possibilities of production in many industrial branches. Additive manufacturing, compared to subtractive manufacturing, brings higher freedom of design and, thus, the ability to produce more complex parts. On the other hand, there is still a lack of standards for heat treatment, non-destructive testing, surface modifications, etc., which are common for conventionally produced parts. In the early stage of technology, the application was restricted to rapid prototyping [1] of parts solely made of plastics and polymers, using Stereolithography (SL) [2] and Fused Deposition Modeling (FDM) methods [3]. A significant development was reached mainly in the case of creating the components from a large scale of commonly used materials, not excluding metals and their alloys in various combinations, using 3D printing [4]. Additive manufacturing also expresses technologies using the welding techniques that are used traditionally for the joining of separate parts. A principle of such Wire Arc Additive Manufacturing (WAAM) technologies lies in the deposition of material in the form of wire, which creates 3D components layer by layer [5]. A specific standard, with the label ISO/ASTM 52900:2021 [6], includes the Selective Laser Melting (SLM) method into the group of technologies, during which the metal in the form of powder, with desired composition, is fully molten in the set thickness, using the laser power. After its cooling, another layer of powder is applied to the workspace, it is molten again, and repeating this process, part of a required shape and size is created from an STL file based on CAD design, using the selected laser scanning strategy [7]. During the last period of using additive manufacturing and the effort of its application in the engineering practice, significant defects occurring in the field of metal components creation, using not only the SLM process but additive manufacturing generally, were defined. Reasons for their presence can be related to the setting of process parameters, powder material properties and conditions, environmental factors, experiences of operating staff, etc. [8,9]. For example, these defects include mainly porosity [10], cracks [11], delamination [12], and many others. Another important parameter, which cannot be identified visually, is the stress state of the material after the AM process. Residual stresses (RS), which are present in all materials after processing, are a significant part of the material surface integrity [13]. It is well known that their compressive character can increase the material’s strength or toughness and vice versa; their tensile character negatively influences the lifetime of the final component, such as causing its fracture, as the consequence of the crack growth acceleration [14]. Due to this important influence on final component behavior, their identification is very important.

Considering that the material is exposed to laser influence during the SLM process, which focuses its high energy density on the small point, stress induction mechanisms can be predicted similarly to common laser processes, such as laser welding. Hence, actuating residual stress mechanisms are sharp thermal gradients situated surrounding the point of the laser impact. Whereas the cumulated heat in the mentioned areas is significantly high, ordinary materials with a certain thermal conductivity are not able to absorb it. As a consequence, the expansion of the surface occurs, which is restricted by subsurface layers, so compressive stresses arise. After reaching the material’s yield strength, plastic deformation in the surface layer occurs. It tends to shrink under the influence of graduate cooling; however, this mechanism is again restricted by subsurface layers; hence, final residual stresses are tensile in the surface layers, and deeper into the material, they are transformed into the compressive character [15]. Taking the magnitude of stresses into account, the important factors are SLM process parameters, which increase or decrease their values. It includes mainly laser energy input, which is affected by the laser power, scan speed, or layer thickness [16,17,18]. Also, the laser scanning strategy turned out to be significant, which is confirmed by the increasing number of publications devoted to this parameter [19,20,21]. An earlier study, made by Mercelis and Kruth [22], mentions the influence of the number of layers on the residual stress state of SLM components. Material properties play a role, too, which was investigated during the research conducted by Vrancken et al. [23]. Printed parts, which are subjected to the influence of bending phenomena [24], mainly after the removal of the substrate, can cause a variation in the residual stress state distribution; hence, this factor also needs to be taken into account.

Non-destructive testing provides solutions for the identification of residual stress states in engineering components, with the main key being positive, related to the inoffensive influence on the evaluated material during its testing. This field includes a few of the most used methods, whose foundations have been laid in the last century. However, their application and optimization are points of interest even within actual research. X-ray diffraction (XRD), whose principle lies in the interaction of the X-ray radiation waves with the crystalline lattice of materials, is the most commonly used technology. Interplanar spacing within the material is influenced by the residual stress variation, and consequently, variation in the diffracted waves occurs. Generally, a penetration depth reaches tens of micrometers [25]. The acoustic–elastic effect, representing the variation in the ultrasonic wave as the reaction to the influence of elastic strain on the material, is fundamental to the ultrasonic testing application for residual stress measurement. As the higher sensitivity was found in the case of a longitudinal wave, a longitudinal critically refracted (LCR) wave, whose penetration depth depends on the frequency and propagates along the surface layer of material, is a frequently used method [26]. The acoustics is also the basics of the acoustic emission method, which shows potential for residual stress measurement [27]. Taking the influence of residual stresses on the magnetic properties of materials, magnetic methods, such as the magnetic strain method, using the magnetostrictive effect, or the Barkhausen noise method, can also be used for residual stress identification [28]. Electromagnetism is the key parameter for the residual stress state evaluation during the application of methods using eddy currents induced into the material [29]. In specific cases where residual stresses are not identified as the parameters of the surface integrity, their identification is made in deeper volumes of materials. Within the application of the acoustic–elastic effect, contactless EMAT transducers provide solutions in the form of ultrasonic birefringence, where two differently oriented waves are used for stress determination [30]. More often, destructive and semi-destructive testing methods are applied, such as deep-hole drilling or contour methods. The first mentioned is the equivalent of the surface drilling method, and it uses a drilling operation in the evaluated area of material, which is trepanned for the relaxation of stresses that are expressed through the comparison of the original and post-relaxation holes. The second applies to the cut of material in the area of interest using the EDM technique, and stresses are evaluated through the FEM of arising contour [31]. In terms of using the mentioned technologies for residual stress identification in additively manufactured products, Chen et al. [32] applied the XRD strategy for the evaluation of residual stresses in aluminum alloy (AlSi10Mg). The fiber optic eddy current sensor was tested for residual stress identification in parts made by subtractive and additive manufacturing [33]. A state-of-the-art review, made by Acevedo and co-authors [34], summarizes the application of the ultrasonic testing method for AM parts RS evaluation. A study focused on the application of laser ultrasound for generating the surface wave describes its application in a laser additive manufactured titanium alloy TC4 for RS identification. The main factors of final stresses were stated in the form of scanning speed, its direction, and the feeding of powder [35]. Other previously mentioned methods were not subjects of some significant research focused on RS in AM materials nowadays. There are some methods for reducing the stress state in the surface layer of printed material related to the AM process, such as re-scanning the surface layer using the laser or heating the substrate platform [36]. Solutions also provide technologies for inducing the compressive stresses in a mechanical way of treatment, such as shot peening or more progressive laser shock and water jet peening methods [37]. However, the stress-relieving heat treatment appears to be the most suitable method for reaching the neutral stress state.

Whereas the heat treatment of additively manufactured 316L stainless steel is not described in some standards, and the material is a little different than the conventionally made one, there was an effort of some researchers in recent years to find the optimal conditions for stress relief in the mentioned material. Within this experiment, focused on microstructure, residual stress, and mechanical properties evaluation, a muffle furnace with argon atmosphere was used, and specimens (made by Selective Laser Melting) were air cooled. Three temperature regimes were chosen for residual stress investigation. At 400 °C for 4 h, 23% stress relief was reached; at 650 °C for 2 h, 65% stress relief was reached, and at 1100 °C for 5 min, a reduction was even to 90%. However, it needs to be mentioned that at such high temperatures, columnar grain growth and coarsening of nano-inclusions occurred [38]. These values confirmed the earlier experiment, devoted to the effect of residual stress on the AM 316L corrosion, carried out by the same authors in the same conditions, where residual stress relief was similar with low deviations, equal to 31.3%, 67.4%, and 93.2% [39]. During the experiment performed by Santa-Aho et al. [40], heat treatment was used as one of the post-processing technologies on L-PBF 316L stainless steel. Stress relief annealing was performed under vacuum at 1030 °C for 1 h, using flowing nitrogen gas cooling, with the consequence in the form of the surface (top and bottom) residual stress decreasing. SLM 316L stainless steel was the subject of interest during this research, which focused on the evaluation of its microstructure and corrosion behavior after the heat treatment. Two temperature regimes were chosen. Specifically, 650 °C and 1050 °C, with 30 min rising to the temperature and its holding for 30 min in Argon atmosphere, with a furnace cooling. A 650 °C regime was defined as stress relieving, which was confirmed by unchanged structure in comparison with 1050 °C [41]. Similar characteristics of the stress relieving regime were described in the study, where the behavior of stress corrosion crack growth in AM 316L stainless steel was investigated. The mentioned stress-relieving temperature regime was set at 650 °C for 2 h in the Argon atmosphere [42]. Stress relieving temperatures of values 400 °C, 800 °C, and 1200 °C were applied to L-PBF 316L stainless steel, and their influence on the strengthening mechanism of the material was investigated during the research made by Riabov et al. [43].

The design of the engineering components is customized not only to functionality, but actual requirements also include economic aspects in the form of input material saving, operation times shortening, or reducing the number of used technologies. An additive manufacturing process shows potential to be an adequate tool for such a manufacturing model. The freedom in the design of unconventional shapes and reaching the quality of 3D printed models contributes to its predispositions. Heat treatment of AM stainless steel is a highly discussed topic, mainly in the case of reaching the stress-relieved state. The aim of the described experiment is to contribute to the issue and help to advance similar research. The influence of applied temperature and holding times on residual stress and microstructure is evaluated, and the effect of the bending phenomenon is also considered. A primary task is to obtain input data for a follow-up experiment focused on setting the optimal parameters of stress-relieving heat treatment in AM stainless steel in order to obtain reference stress-free specimens for eddy current testing identification of residual stresses in a surface layer of evaluated material.

## 2. Materials and Methods

### 2.1. Experimental Specimens Preparation

A basic specimen with dimensions 140 × 30 × 10 mm was prepared by Selective Laser Melting (SLM) technology at the Technical University in Ostrava (Czech Republic)—Center of 3D printing Protolab. The system used for printing was RenAM500S Flex by Renishaw company (Wotton-under-Edge, UK), which provides laser power up to 500 W and a scanning speed of 2000 mm·s^−1^. This system allows for making specimens in the chamber with dimensions 250 × 250 × 350 mm and the possibility of printing speed in the range of 5 to 20 cm^3^·h^−1^. One of the most commonly applied materials for 3D printing was SS 316L stainless steel powder, which was also selected for this experiment by the same manufacturer—Renishaw (Wotton-under-Edge, UK). Its composition [44] is summarized in Table 1, and the printing parameters are included in Table 2.

Energy input was calculated based on the next Equation (1) [45].

*Ε* = *P*/(*v* × *d* × *t_L_*) (1)

**Figure 1:**
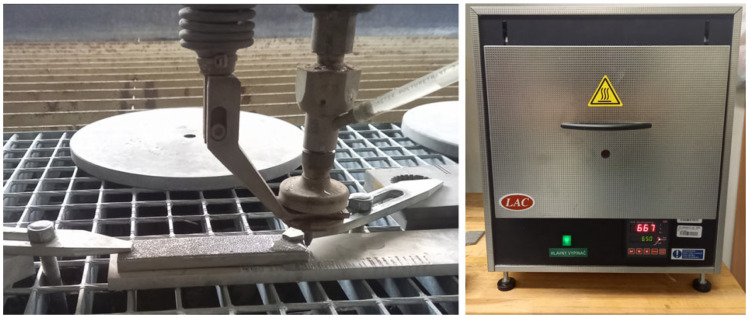
AWJ cutting of specimens and muffle laboratory furnace LAC, type LMH 07/12.

After the printing process, a part was cut into 14 pieces, with dimensions of 10 × 15 × 10 mm, using the Abrasive Water Jet (AWJ) machine Water Jet 3015 RT-3D by PTV company (Hostivice, Czech Republic), for its negligible influence on the stress state of the material, in the abrasive water jet laboratory of Faculty of Manufacturing technologies in Prešov (Figure 1). All cuts were made at the high pressure (400 MPa), produced by PTV JETS—3.8/60 pump (PTV, Hostivice, Czech Republic) and in the middle (Q3) quality according to SN 214001:2010 standard [46], which corresponds to a velocity equal to 164.22 mm·min^−1^. The mentioned standard defines 5 qualities of reached cut surface, by which Q1 is the lowest and Q5 is the highest. Based on a selected quality, cut velocity is calculated by the machine. Abrasive (Australian Garnet—MESH 80) flow was set on a value of 400 g·min^−1^. The water nozzle diameter was 0.3 mm, and the focusing tube diameter was 0.9 mm. A nozzle’s standoff distance of the specimen was permanently set at 4 mm. For the microstructure comparison, conventionally made AISI 316L specimen 0 was included in this experiment.

### 2.2. Heat Treatment

The heat treatment was run in the muffle laboratory furnace (LAC, type LMH 07/12) by LAC company (Židlochovice, Czech Republic), with the chamber in dimensions 170 × 170 × 275 mm (Figure 1), in a Faculty of Manufacturing Technologies in Prešov. Specimens were divided into 4 groups based on temperature (650 °C, 700 °C, 750 °C, 800 °C), which was selected based on results of studied publications from the field in the range of predicted absence of significant microstructural changes. For all specimens, the rising time to the required temperature was set at 70 min, which corresponded to a heating rate equal to 9.29, 10, 10.71, and 11.43 °C·min^−1^ for the mentioned temperatures. For all four temperatures, specimens were held for 4, 6, and 8 h. After reaching the desired temperature and the required time, specimens were cooled in the still air. Specimen 1 from the side and specimen 8 from the middle of the original part were not heat-treated for inspection of the influence of the bending phenomenon of a part after the removal of the 3D printer substrate.

### 2.3. X-ray Diffraction

For the residual stress state evaluation within the specimens, X-ray diffraction was performed on their surface. This partial experiment was performed in the Department of Machining and Manufacturing Technologies at the University of Žilina. The used machine was Proto iXRD with MG40 goniometer by Proto company (Wrocław, Poland), and the sin^2^ψ plane reflection method was carried out. During such measurements, XRD was repeated at certain tilts of the ψ angle, and spacing between crystallographic planes was measured. Angle tilts and spacing were included in the graph, and the slope of the curve, expressed by gradient, was fundamental for the residual stress calculation, based on Equation (2), taking the elastic characteristic of the material into account [47].

**Figure 2:**
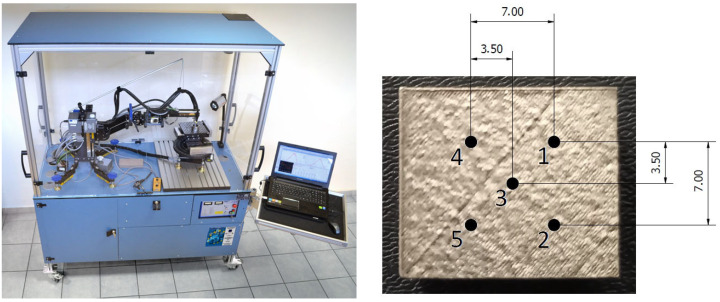
Proto iXRD machine with measurement points on each specimen.

*σ* = (*E*/(1 + *υ*)) × *m* (2)

where *σ* is the residual stress; *m* is the gradient of the curve, and *E* (Elastic modulus) and *υ* (Poisson’s ratio) are the material’s properties. Taking the parameter settings into account, the Mn_K (α) X-ray tube was used, with a collimator of diameter of 1 mm and a Cr filter. The input voltage was equal to 20 kV, and the current was 4 mA. Beta (β) oscillation was 3°, and 15 angle positions were performed on one measurement point (±30°). A penetration depth at these conditions is approximately 10 μm. Each specimen was subjected to 5 measurements, of which 4 measurements were performed in the corners and 1 in the middle of specimens (Figure 2), on their top surface. In the case of all 14 specimens, overall, 70 measurements were carried out.

### 2.4. Microstructure Evaluation

Evaluation of the microstructure after the heat treatment was performed by the Department of Materials and Technologies for Vehicles at the Technical University in Ostrava and included several partial measurements. Namely, pycnometric density and average chemical composition determination, OM, and SEM/EDX analysis. For pycnometric density determination, a He pycnometer AccuPyc II 1340 by Micromeritics company (Norcross, GA, USA) was used, and the relative density of specimens was calculated according to the next Equation (3) [48]:

*ρ_rel_* = (*ρ_pyc_*/*ρ_teo_*) × 100 (3)

where *ρ_pyc_* refers to the determined pycnometric density of specimens; *ρ_teo_* refers to the theoretical density (set according to published values between 7.95 and 8.00 g∙cm^−3^ [40]) of a default specimen 0 (conventionally made AISI 316L), and *ρ_rel_* is the relative density. An average chemical composition of specimens was obtained using the EDS method, which states the composition as the arithmetic mean of 3 independent area measurements, in size of approximately 1 mm^2^. A device used for this measurement was a scanning electron microscope QUANTA 450 FEG by FEI company (Hillsboro, OR, USA), which is equipped with the EDAX APOLLO X microprobe (Ametek, Berwyn, PA, USA) for the energy-dispersive X-ray analysis. The same device was used for the microstructure imaging of the secondary electrons (SE) mode. Also, an optical microscope (OM) Olympus GX 51 by Olympus company (Center Valley, PA, USA) was used. To secure the quality of obtained data, specimens were embedded into Polyfast resin and metallographically prepared using wet grinding with SiC paper and polished using the Al_2_O_3_ suspension. For better depiction, evaluated microstructures were highlighted by the application of etching with Carpenter reagent.

## 3. Results

### 3.1. Residual Stresses—XRD

X-ray diffraction was performed on five points on the surface of each experimental specimen. Normal stresses, which express stress state in materials and are major for residual stress expression, were monitored. If the value is positive, stresses are tensile, and vice versa—if the value is negative, the stress state is compressive. In the case of values closest to zero, the stress state can be considered neutral (relieved stress state) in a depth of interest. Table 3 summarizes these values. Stress values of five points within each specimen were average (Avg.), maximum (Max.), and minimum (Min.); measured values were chosen, and the absolute value, closest to zero (|Min.|), within each specimen was expressed. Despite the fact that |Min.| values are absolute, the sign is expressed because of distinguishing between the tensile and compressive character of the stress state. According to assumptions, the highest value of stresses was measured on the first specimen, which was not heat-treated. The average value in this specimen is 119.7 MPa; the maximum value (157.5 MPa) was reached at point 1, and the value closest to zero was in its middle, which is also a minimum stress value in the specimen (93.7 MPa).

In the case of 650 °C heat treatment temperature, the closest value to zero was reached in specimen 3 (−2.2 MPa), specifically at point 2. However, taking the average value into account, the most neutral stress state was reached in specimen 4 (−6.7 MPa). Kong et al. [41] performed an experiment focused on heat treatment on the same material, where a 650 °C temperature regime for 30 min was defined as stress relieving. Similarly, in another study, the same statement was given [42], but with a longer duration of 2 h. The potential of the mentioned temperature for stress relief was confirmed within this experiment but with a longer duration of heat treatment and application of three-time intervals. Table 3 shows that 4-h holding at this temperature (specimen 2) was not so effective, and slight stress relieving was reached. Stress states were decreased, but they still had significant tensile character. The 6 and 8-hour durations reached more auspicious results, which is proof that it is more appropriate to use longer heat treatment at 650 °C. The reason for the difference between the mentioned studies can be the absence of a protective atmosphere during this experiment. The change in the residual stress character with increasing time is presented in Table 3, which cannot be reached by relieving heat treatment. The reason for such a phenomenon is the original stress state of the specimen. Specimens closer to the edge of the original part tend to include tensile stresses, and specimens closer to its middle tend to include compressive stresses, which is the result of the bending phenomenon described below. Hence, heat treatment suppressed residual stresses from higher tensile or compressive values based on the original position of each specimen. Similar behavior was also present during other testing temperatures.

Within the experiment by Cruz et al. [39], including the residual stress relief under heat treatment of additively manufactured stainless steel 316L, it was found that increasing the temperature caused higher stress relief when the used temperatures were 400 °C, 650 °C, and 1100 °C. The next paragraphs confirm this phenomenon, but its influence was shown in the range of temperatures, which are predicted below the occurrence of significant microstructure changes, and moreover, the similar influence of holding times was proven.

Specimens 5, 6, and 7 were heat-treated at 700 °C temperature. In the specimen 5 (4 h), only compressive stress states were reached in all measured points. The highest compressive stresses were at point 5, equal to −69 MPa, and the lowest and the closest to zero stress state was at point 1, equal to −5 MPa. For 700 °C temperature, the highest tensile stresses were present at point 5 for specimen 6 (43.9 MPa). Specimen 7 reached the most neutral stress state of all specimens (0.4 MPa average value). Moreover, maximum and minimum values were among the most neutral, 11.3 MPa and −18.1 MPa, respectively.

Specimen 8 was also not heat-treated. Despite the fact that specimens 1 and 8 were not heat-treated, a significant difference in stress character was observed (specimen 1: 119.7 MPa and specimen 8: −20.46 MPa). The reason for this is the bending phenomenon of the part after its removal from the substrate following the printing process.

Within the third temperature regime (750 °C), it can be stated that neutral stress values (Avg.) were reached, except for specimen 11. Obtained values of RS were −5.92 MPa for 4 h (specimen 9) and 1.62 MPa for 6 h (specimen 10), respectively. In specimen 10, the most neutral value (0.4 MPa) was present at the middle point 3. Specimen 11 includes tensile stresses (33.74 MPa) with a maximum value (62.5 MPa) at point 4.

The same phenomenon occurred within the specimens, which were heat-treated at 800 °C, but with a difference that specimen 12 (the first of three specimens within this regime) reached the highest deviation of neutral stress state (Avg. 20.56 MPa and Max. 31.2 MPa). This makes sense if the holding time is taken into account. A period of 4 h is shorter; hence, the specimen is not exposed to temperature influence for sufficient duration, and consequently, stresses are not relieved to such an extent. For the other two specimens (13 and 14), the average values were 1.44 MPa and −6.46 MPa, respectively. The previously-mentioned influence of the increasing temperature was confirmed within the experiment of Chao et al. [38], but besides the stress relief, it was found that microstructure changes could occur at elevated temperatures, mainly in the form of the inclusion precipitation, which can be present in the case of the used temperature regimes in this study. Considering that lower temperatures were chosen, slighter microstructure variations can be predicted.

**Figure 3:**
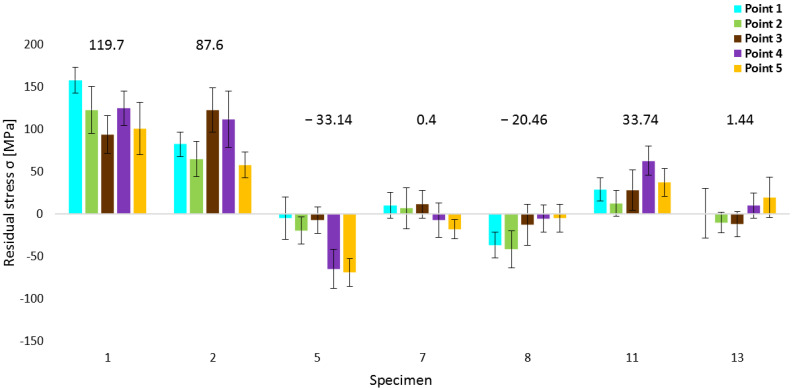
Residual stresses in chosen experimental specimens.

Selected residual stress values are plotted in the next Figure 3. The first specimen was selected for its highest average residual stress value. Considering the fact that this specimen was not heat-treated, it can be the reference for comparison with other specimens that were subjected to the mentioned process. The second specimen value shows unsatisfactory results for stress relieving, using the combination of the lowest experimental temperature (650 °C) and short duration (4 h) of the heat treatment. In the case of the 700 °C regime, two significant phenomena occurred. At 4-hour duration (specimen 5), the lowest average residual stresses were reached, equal to −33.14 MPa, and at 8-hour duration (specimen 7), the most relived state was reached, equal to 0.4 MPa. It is important to mention that specimen 7 is from the middle of the original part, the same as the specimen 8. Therefore, heat treatment relieved stresses from lower values, possibly similar to specimen 8 (−20.46 MPa), than in the case of the specimens on both sides of the original part. Although specimen 11 was heat-treated for the longest duration (8 h) during the 750 °C temperature regime, its value exhibited the highest deviation from the relieved state in comparison with specimens 9 and 10. The position of specimen 11 was close to the right side of the original part; thus, the initial residual stress value was higher, which consequently led to a lower neutral final stress state. Finally, from the last three specimens (12, 13, 14), heat-treated at 800 °C, specimen 13 was selected for its most neutral stress state. However, reaching the microstructure after heat treatment can be decisive in the choosing of the most appropriate temperature regime.

### 3.2. Microstructure Evaluation

The mentioned selected specimens were included in the microstructure evaluation. The next table (Table 4) contains the resulting data of pycnometric density determination, where specimen 0 (AISI 316L) represents the reference (100%) density. Any of the additively manufactured specimens did not reach full density, which confirms the fact that 100% density is difficult to reach after powder bed fusion processes. In this experiment, at the highest temperature (800 °C—specimen 13), the lowest density (99.6882%) was reached, and for the comparison, the highest relative density value (99.8109%) was in the case of specimen 2, where 650 °C temperature regime was used. Shin et al. [49] found that the heat treatment increased the density of the material (at 1100 °C), which does not match our results. A reason can be the lower temperature range of used regimes in this experiment, which has a lower influence on the microstructural variations occurrence. The resulting density values can be considered satisfactory, as the lowest value did not decrease below 99.6%. Calculations for other specimens showed relative density between these two values.

The next table (Table 5) includes the results of the selected specimens’ average chemical composition determination. Iron (Fe) content within each specimen is in the range of 64–66%. Chromium (Cr) is included at lower values, between 16–17%, in comparison with the chemical composition stated by the powder manufacturer (Table 1). In the case of Nickel (Ni), its content within all specimens was approximately 13%, except the conventionally made specimen 0, which reached a lower value (9.9%). Molybdenum (Mo) content reached values closer to the high limit (approximately 3%) in all specimens. Manganese (Mn) was present in the range of 0.6–1.3%. The exception was again specimen 0, where Mn content was a little higher, equal to 3.2%. Silicon (Si) content exceeded the high limit stated by the powder manufacturer in almost all specimens. In general, it can be stated that all specimens made by additive manufacturing matched the chemical composition stated in a material data sheet, with negligible deviations.

**Figure 4:**
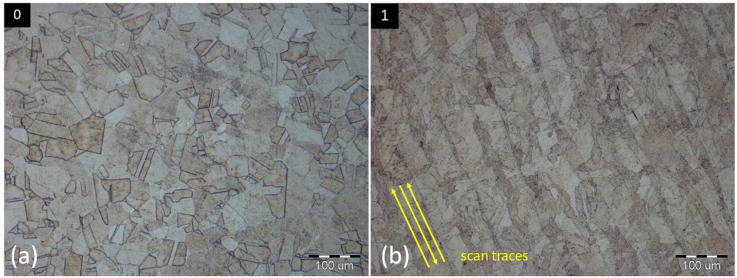
Microstructure of conventionally made specimen 0 (**a**) and SLM untreated specimen 1 (**b**).

**Figure 5:**
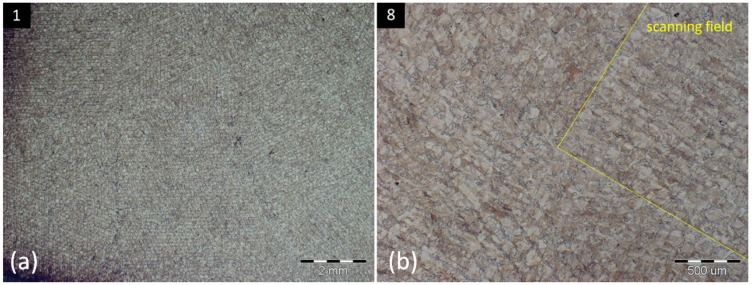
Scanning field interfaces within the Chessboard scanning strategy in specimens 1 (**a**) and 8 (**b**).

As can be predicted, Figure 4a of specimen 0 resembles the typical microstructure of conventionally made AISI 316L stainless steel, with austenitic equixially variously oriented grains in sizes from 10 to 100 μm. In comparison, the microstructure of specimen 1 is typical for the material made by 3D printing, using the Selective Laser Melting process, which includes irregular grains (sizes from 20 to 50 μm) with various orientations. It can be ascribed to the influence of laser energy, rapid cooling, and bonding of the individual layers within the selected scanning strategy during the printing process. Characteristic indicators are elongated grains in the direction of scan traces, resembling the sloping lines, as shown in Figure 4b. The orientation of the mentioned scan traces is dependent on the rotation angle of each field within the Chessboard scanning strategy. Interfaces of these fields can be seen in specimens 1 (Figure 5a) or 8 (Figure 5b).

**Figure 6:**
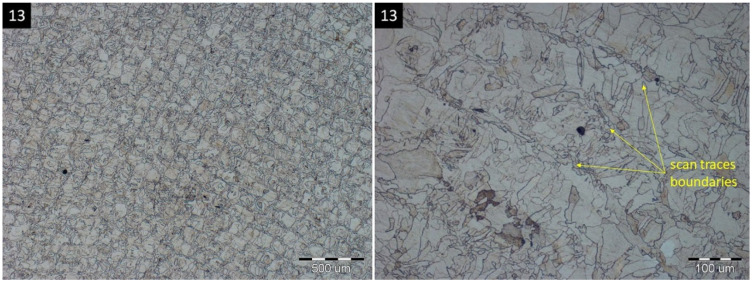
Austenitic grains within the scan trace boundaries in specimen 13.

A specific scanning process within the SLM also has an influence on the size of grains, according to their position within the scan traces. Grains present inside scan traces reach larger sizes, up to 50 μm, while the influence of two adjacent scan traces causes the creation of smaller grains, approximately 20 μm, present on the edges of the scan traces. This phenomenon is interpreted in Figure 6.

**Figure 7:**
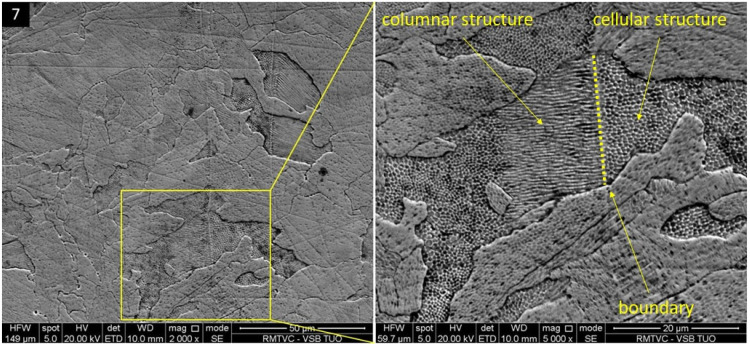
Cellular structure of grains in a specimen 7.

Grains themselves, within all specimens, consist of the texture, characteristic of the Selective Laser Melting process, in the form of fine cellular structure with various orientations (Figure 7), whose arising mechanism and associated mechanisms are still comprehensively unexplained [50]. The assumed formation of such fine structure can lie in the solidification process within the SLM, characteristic of a faster cooling rate associated with more significant undercooling. Such a cooling rate causes a restriction of time needed for secondary dendrite development. [51] Another scenario is described in the case of the presence of these structures on the boundary of grains as the consequence of rapid cooling, causing the formation of segregates on the mentioned boundary in the form of cellular structure [52]. It was found that the conversion between cellular and reoriented (columnar) structures occurs naturally in the form of morphology variation, as on the melt pool interfaces in the case of grain boundaries [53].

**Figure 8:**
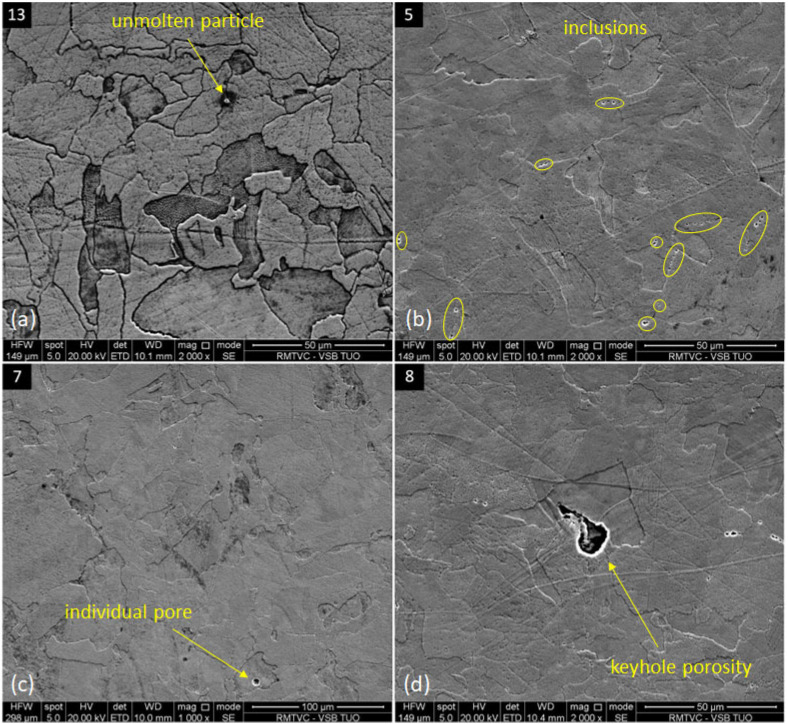
Discontinuities in specimens: (**a**)—unmolten particle—13; (**b**)—inclusions—5; (**c**)—individual pore—7; and (**d**)—keyhole porosity—8.

Specimens made by additive manufacturing included commonly occurring discontinuities related to the SLM technology. A negligible number of identified powder particles were randomly entrapped within the structure as a consequence of insufficient melting (Figure 8a). Inclusions on the grain boundaries were found within all evaluated specimens, and the example of specimen 5, which was heat-treated, is shown in Figure 8b and in Figure 8d, which was evaluated in an as-printed state. The origin of the mentioned inclusions is the precipitation of two possible phases. The first scenario is described within this research [54], where δ-ferrite was identified in the case of an as-printed state and in the heat-treated specimens, resulting from the multiple preheating processes. The presence of content of chromium and molybdenum elements is also associated with this precipitation for their ferrite-creation character, with the conclusion that heat treatment did not influence this phase composition. In another study [38], it was described that inclusions are of σ-phase nature, resulting from its precipitation on austenitic grains between 650 °C and 800 °C (the same temperature range is used in this research) in the form of its precursors at a lower temperature and its precipitation itself and embrittlement at a higher temperature. The first occurrence of δ-ferrite was identified at 1400 °C. The powder composition and other SLM process factors influence the character of these inclusions, and their analysis is worth a more extensive study. A smaller portion of porosity was identified during higher magnification, sporadically in the form of individual pores generally in the size of up to 20 μm, mainly with random occurrence within the microstructure. A type of such porosity was predominantly in the oval shape, caused by the gas entrapment during the local melting of the material (Figure 8c). Another type was the specific keyhole porosity in the larger size, which is caused by the local layer collapse (Figure 8d).

**Figure 9:**
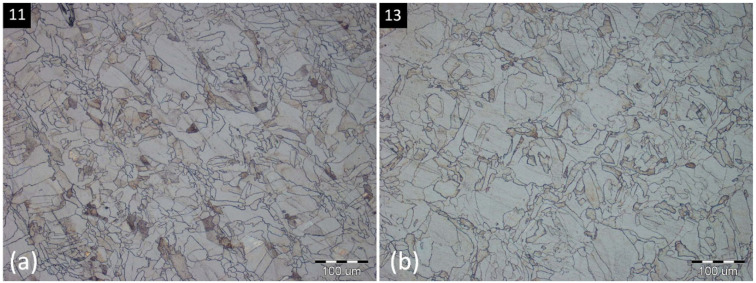
Microstructure after the heat treatment: (**a**)—specimen 11 (750 °C, 8 h); (**b**)—specimen 13 (800 °C, 6 h).

Heat treatment caused some variations within the microstructure of evaluated specimens. No significant differences were found in the case of the 650 °C temperature regime; however, the first changes occurred already at 700 °C, where the markedness of scan traces was reduced with the increasing of applied temperature. Furthermore, with the temperature rising, austenitic grain boundaries are more significant, which can be the partial consequence of graduating inclusion precipitation in these areas, and the etching process plays its role, too. At 750 °C (specimen 11—Figure 9a) and 800 °C (specimen 13—Figure 9b), further grain growth is present, mainly in the case of the second-mentioned specimen, reaching sizes of up to 100 μm.

It can be stated that some microstructure variations occurred during the heat treatment of chosen experimental specimens; however, no significant recrystallization process was present because of the presence of fine austenitic grains, characteristic of this 3D printed material. However, the mentioned variations could cause a variation in the eddy current testing signal, which could be crucial for the application of this technology for residual stress identification.

### 3.3. Effect of Heat Treatment on Residual Stresses

**Figure 10:**
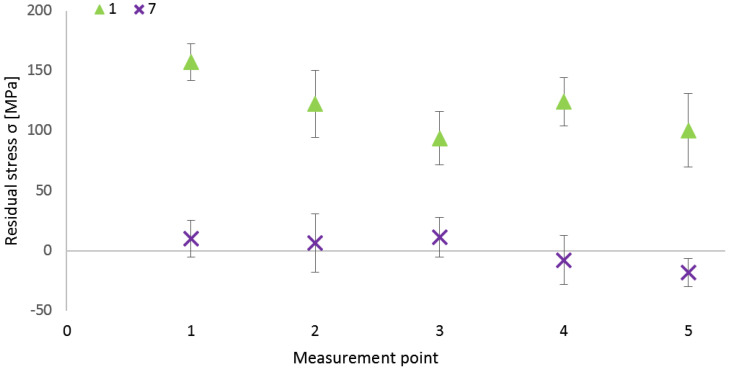
Residual stresses in specimens 1 and 7.

According to diffraction resulting data, the most neutral stress state after the stress relaxation heat treatment process was reached during the processing of specimen 7, specifically, at a 700 °C temperature regime of an 8-hour holding. The resulting average residual stress is 0.4 MPa, with a maximum (tensile) stress at point 3 equal to 11.3 MPa, minimum (compressive) stress at point 5 equal to −18.1 MPa, and the most neutral value (6.4 MPa) at point 2. Figure 10 includes a comparison of specimen 1 and specimen 7. It compares the magnitude of residual stresses in every single point of the measurement (see Figure 2) within both specimens. The measured values of specimen 7 are situated around the central horizontal line, corresponding to the neutral residual stress state. Taking the measurement variance into account, it is quite similar for all measurements and both specimens. A variance, or a statistical error, refers to the uniformity of stress distribution in the evaluated point. The lower the value of this variance (also relative to the normal stress value), the more homogenous the material and the more the stress uniformity. The highest variance was reached in the case of point 5 (±30.8) in specimen 1, and the lowest was reached at point 5 (±11.5) in specimen 7. Such values of the variance were reached during all 70 measurements of specimens. If the whole area (comparison of all five points) is the point of interest, higher deviations are reached in specimen 1. The difference between the maximum (Max. = 157.5 MPa) and minimum (Min. = 93.7 MPa) values is 63.8 MPa, whereas in specimen 7, this difference is 29.4 MPa (Max. = 11.3; Min. = −18.1). This confirms the fact that the heat treatment caused a neutral residual stress state in specimen 7 and also caused a more balanced stress state between all five measurement points, which is proof of reaching the stable stress state within the mentioned heat-treated specimen. Also, it can be stated that the number of measuring points within one specimen needs to be adapted to its size based on various residual stress values between all points.

### 3.4. Effect of Bending Phenomenon

**Figure 11:**
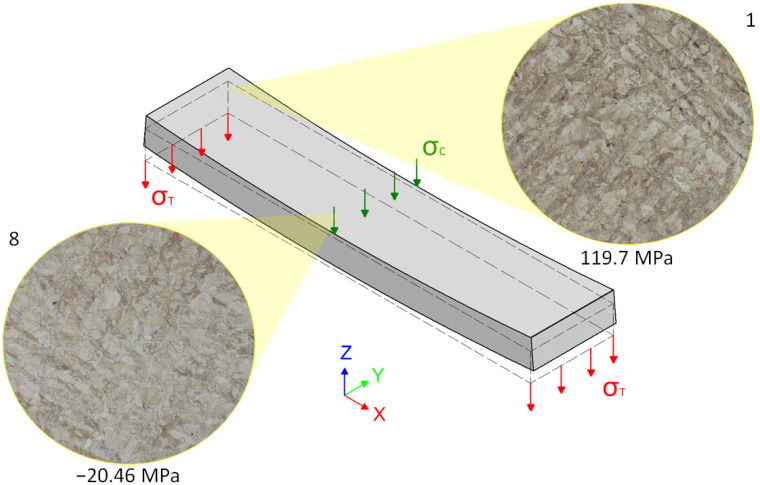
Residual stress distribution after the bending of SLM part (σ_T_—tensile stress; σ_C_—compressive stress) [24].

A comparison of residual stresses between specimens 1 and 8 is also worth mentioning, despite the fact that both specimens were not heat-treated and their microstructures were identical. It can be expected that these two specimens should include the same or at least similar stress states. The bending phenomenon, occurring in the case of longer specimens, was described in the research performed by Liu, Yang, and Wang [24], which pointed to its impact on the shape and deformation of the component. Similar bending of an original part after the removal of a substrate and its impact on the residual stress distribution need to be considered. It occurs as the consequence of parts dimensions, specifically, the ratio between the length of edges in the x and y axes, in combination with a quite small thickness, despite using the support structures. The part is under the influence of tensile strain before it is removed from the platform. Right after the removal process, these strains are relaxed, which leads to the bending under a certain angle of the part. During this process, the top surface layer is compressed in its middle area, which consequently leads to the induction of compressive residual stresses. On the opposite bottom surface, the part is stretched, which leads to the lifting of both sides on the longer (x) axis in a z-axis direction. The consequence is resulting in tensile residual stress on both sides. Such a residual stress distribution within the original part is plotted in Figure 11. Taking a diffraction resulting data into account, this phenomenon is confirmed. In specimen 1, from the left side of a part, the average residual stress is equal to 119.7 MPa, whereas in specimen 8, from the middle of the part, the average residual stress is equal to −20.46 MPa. It makes a difference in residual stress between these specimens and also between the middle and left sides of the part, equal to 140.16 MPa.

### 3.5. Effect of Important Factors on Stress Relief

XRD stress measurement results showed that besides temperature and time, the original stress state and the shape of the specimen need to be taken into account for appropriate heat treatment process settings. A combination of 650 °C temperature and 4-hour holding reached unsatisfactory results. In the case of 6 and 8 h, stress values approached the neutral state. However, in the case of larger sizes of specimens, the mentioned temperature might not be able to relieve residual stresses to such an extent. In comparison, the 700 °C temperature regime reached better results. During 4 h, the least neutral state was reached. In comparison with 650 °C at the same duration, this value is more satisfactory. However, it needs to be mentioned that specimen 5 is closer to the middle of the part, where more compressive stresses are present due to the bending phenomenon. For 6 and 8 h, it can be stated that the reached values represent a relieved state, but again, the positions of specimens 6 and 7 play it role, mainly in the case of specimen 7, which is straight in the middle of the original part. After the heat treatment at 750 °C, a similar phenomenon occurred. Even here, the 4-hour heat treatment reached quite a neutral stress state. Again, the positions of specimens 9 and 10 need to be mentioned. During the 8-h heat treatment, the least neutral stress state was reached at this temperature regime as the consequence of specimen 11 positions, situated closer to the right side of the part, where more tensile residual stresses were present. At the 800 °C heat treatment, the neutral values were reached all three times. During 4 h, the highest residual stress value was reached, which confirmed previous claims about the short-time influence. For 6 and 8 h, it can be stated that relieved stresses are present. However, at such high temperatures, slight microstructure changes can occur.

## 4. Discussion

After the processing of the measured data, the similarities with other experiments from the field can be found with some new contributions of this research to the issue. Previously mentioned studies [38,39] described increasing temperature as an important parameter causing greater stress relaxation in the AM 316L stainless steel. This experiment confirmed the statement, but heat treatment was applied at a greater number of temperatures in the range of slight microstructure changes. Moreover, the duration of heat treatment within all temperature regimes was monitored, with a similar effect on relaxation. In publications [41,42], 650 °C temperature is defined as stress-relieving for 30 min and 2-h heat treatment, respectively. A 650 °C temperature was monitored for 4, 6, and 8 h during this experiment, and the results showed low potential for a shorter duration of the heat treatment at this temperature regime. The absence of a protective atmosphere in a muffle furnace could be the main factor of this finding. The presented study provides microstructure analysis at lower applied temperatures of heat treatment, where slight changes occurred, based on which it can be stated that such heat treatment has the potential for the creation of reference stress-relieved AM 316L specimen for residual stress identification using eddy current testing method. The bending phenomenon, occurring in the case of longer specimens after 3D printing [24], has an obvious influence on the character of residual stresses in the surface layer of components. Their magnitude, which can be crucial for setting the parameters of the post-processing operations, such as heat treatment, was stated during the preliminary analysis of samples 1 and 8. For residual stress identification, the XRD method was used, which is limited to a few microns. Hence, the obtained data are related to the surface layer of the evaluated material, which is sufficient for such research, considering the application of the eddy current testing method, which is intended for the surface layers of the materials. Measured relative density is worth mentioning and needs to be monitored more extensively because of its decrease during heat treatment, whereas other studies from the field [49] describe the opposite effect. A reason can be the lower temperature range of used regimes in this experiment, which has a lower influence on microstructural variations occurrence. Based on the presented results, a study can serve as the base for follow-up research focused on setting the appropriate conditions of SS 316L material heat treatment, taking the microstructure variations into account. Considering the application of the lower temperatures with slight microstructure variations, these results are an important stepping stone for setting heat treatment parameters to create the stress-free reference specimen for the eddy current testing identification of residual stresses in AM 316L stainless steel surface.

## 5. Conclusions

The described experiment is focused on the heat treatment of SS 316L stainless steel made by the Selective Laser Melting process, whose purpose was to evaluate the influence of chosen temperature regimes to obtain the material, including neutral residual stress state in its surface layer as the potential reference specimen for eddy current testing of surface residual stresses. Combinations of four temperatures and three holding times were selected, and their influence on residual stress state in surface layers of specimens was analyzed, taking the microstructure variations and related circumstances into account, with the following conclusions:

- The resulting values of residual stresses in the surface layer of experimental specimens, obtained using the X-ray diffraction, showed the potential of used temperature regimes for residual stress relieving in these areas of SS 316L stainless steel and also used muffle furnace proved to be a suitable tool for this post-processing operation;
- The combination of the lower temperature (650 °C) and shorter duration of heat treatment (4 h) seemed to be less effective, according to the fact that measured residual stresses showed higher tensile character;
- The application of the highest temperature of used regimes was shown to be the reliable parameter for the stress state relieving process, mainly in the case of using longer holding times (6 and 8 h). However, it is worth mentioning that some microstructure changes occur during the application of higher temperatures, but recrystallization is not so significant;
- The microstructure of specimens, made by the SLM process, includes irregular grains with fine cellular structure, and any variations were not observed in this type of structure within all specimens. However, the influence of heat treatment was found on characteristic texture with scan traces, whose markedness was suppressed already at 700 °C. The most important microstructure difference was in the case of the highest temperature (800 °C), where slight grain growth occurred;
- The stress state closest to zero was reached in specimen 7; however, it needs to be considered that the used temperature regime suppressed stresses of lower values, closer to zero, which was the consequence of the described bending phenomenon. The position of experimental specimens within the original SLM part was crucial for the stress relieving process, on the basis of what can be stated that the original stress state of the material is important for setting the optimal parameters of stress relieving heat treatment.

Before the setting of the exact parameters of heat treatment for obtaining the neutral stress state specimen as the reference for checking the potential of the eddy current testing method within the residual stress evaluation, other studies need to be realized to answer the still-open questions. It is important to check the ability of used temperature regimes for stress relieving on specimens of larger sizes. Furthermore, it is worth considering the use of a furnace with a protective atmosphere, mainly for securing the cleanliness of the chamber. It would be appropriate to check the selected temperature regimes on a larger number of the same specimens, made under the same conditions of the SLM process, and express the percentage of residual stress relieving during individual regimes.

## Figures and Tables

**Table 1:** Chemical composition of SS 316L stainless steel [44].

Element	Fe	Cr	Ni	Mo	Mn	Si	N	O	P	C	S
**Mass (%)**	Balance	16–18	10–14	2–3	≤2	≤1	≤0.1	≤0.1	≤0.045	≤0.03	≤0.03

**Table 2:** SLM process parameters.

Parameter	Symbol	Value	Unit
Laser power	*P*	200	W
Scanning velocity	*v*	650	mm·s^−1^
Hatching distance	*d*	110	μm
Layer thickness	*t_L_*	50	μm
Energy input (1)	*ε*	55.94	J·mm^−1^
Scanning strategy		Chessboard	
Gas protection		Argon (Ar)	

**Table 3:** Residual stresses at various temperature regimes.

Specimen	Temperature *T* [°C]	Time *t* [h]	Heating Rate [°C·min^−1^]	Normal Stress σ [MPa]			
				Avg.	Max.	Min.	|Min.|
1	-	-	-	119.7	157.5	93.7	93.7
2	650	4	9.29	87.6	122.2	57.5	57.5
3		6		−16.84	12.9	−49.7	−2.2
4		8		−6.7	40.8	−28.4	−5.5
5	700	4	10	−33.14	−5	−69	−5
6		6		2.56	43.9	−38.4	10.3
7		8		0.4	11.3	−18.1	6.4
8	-	-	-	−20.46	−5.2	−41.9	−5.2
9	750	4	10.71	−5.92	17.2	−33.1	1.3
10		6		1.62	31.9	−18.2	0.4
11		8		33.74	62.5	12.4	12.4
12	800	4	11.43	20.56	31.2	7.6	7.6
13		6		1.44	19.5	−12.2	0.7
14		8		−6.46	17.5	−41.2	−0.7

**Table 4:** Determined values of relative density in chosen experimental specimens.

Specimen	Pycnometric Density (g∙cm^−3^)	Standard Deviation (g∙cm^−3^)	Relative Density (%)
0	7.9859	0.0061	100
1	7.9701	0.0047	99.8022
2	7.9708	0.0071	99.8109
5	7.9656	0.0069	99.7458
7	7.9653	0.0044	99.7420
8	7.9654	0.0018	99.7432
11	7.9663	0.0020	99.7545
13	7.9610	0.0029	99.6882

**Table 5:** Measured chemical composition of chosen experimental specimens.

Specimen	Chemical Composition—Mass (%)					
	Fe	Cr	Ni	Mo	Mn	Si
0	65.7 ± 0.9	16.0 ± 0.6	9.9 ± 0.7	3.3 ± 0.2	3.2 ± 0.8	1.9 ± 0.2
1	65.3 ± 0.5	16.3 ± 0.5	12.8 ± 0.3	3.1 ± 0.1	1.0 ± 0.3	1.5 ± 0.2
2	64.1 ± 1.0	16.8 ± 0.1	13.5 ± 0.6	3.2 ± 0.4	1.1 ± 0.2	1.3 ± 0.3
5	65.2 ± 1.3	16.8 ± 0.4	13.1 ± 0.3	2.9 ± 0.2	0.7 ± 0.5	1.2 ± 0.1
7	64.4 ± 0.3	16.7 ± 0.9	13.2 ± 0.8	3.3 ± 0.1	1.1 ± 0.3	1.3 ± 0.3
8	66.3 ± 0.2	17.0 ± 0.3	12.8 ± 0.3	2.5 ± 0.3	0.6 ± 0.1	0.9 ± 0.1
11	64.2 ± 1.0	16.6 ± 0.1	13.1 ± 0.4	3.5 ± 0.9	1.3 ± 0.3	1.4 ± 0.4
13	64.5 ± 0.6	17.1 ± 0.6	13.0 ± 0.5	3.4 ± 0.3	0.7 ± 0.4	1.4 ± 0.1

## Data Availability

Not applicable.
